# Modulatory Effects of A1 Milk, A2 Milk, Soy, and Egg Proteins on Gut Microbiota and Fermentation

**DOI:** 10.3390/microorganisms11051194

**Published:** 2023-05-03

**Authors:** Nuomin, Riyang Baek, Takeshi Tsuruta, Naoki Nishino

**Affiliations:** Department of Animal Science, Graduate School of Environmental and Life Science, Okayama University, Okayama 700-8530, Japan; norman@s.okayama-u.ac.jp (N.); tsurutafe@oayama-u.ac.jp (T.T.)

**Keywords:** A1 milk, A2 milk, casein, egg, gut microbiota, mice, protein, soy

## Abstract

Milk can be divided into A1 and A2 types according to β-casein variants, and there is a debate about whether A1 milk consumption exacerbates gut environments. This study examined the cecum microbiota and fermentation in mice fed A1 casein, A2 casein, mixed casein (commercial casein), soy protein isolate, and egg white. The cecum acetic acid concentration was higher, and the relative abundances of Muribaculaceae and Desulfovibrionaceae were greater in mice fed A1 versus A2 casein. The other parameters of cecum fermentation and microbiota composition were similar among the mice fed A1, A2, and mixed caseins. The differences were more distinctive among the three caseins, soy, and egg feedings. Chao 1 and Shannon indices of the cecum microbiota were lowered in egg white-fed mice, and the microbiota of mice fed milk, soy, and egg proteins were separately grouped by principal coordinate analysis. Mice fed the three caseins were characterized by a high abundance of Lactobacillaceae and Clostridiaceae, those fed soy were characterized by Corynebacteriaceae, Muribaculaceae, and Ruminococcaceae, and those fed egg white were characterized by Eggerthellaceae, Rikenellaceae, and Erysipelatoclostridiaceae. Thus, although several differences can arise between A1 and A2 caseins in terms of their modulatory effects on gut environments, the differences between milk, soy, and egg proteins can be more distinctive and are worth further consideration.

## 1. Introduction

The amount of research on gut microbiota has rapidly increased because of its potential for health promotion and disease prevention. Many factors can influence gut microbiota colonization and diversity, including age, genetics, medications, geographical location, and the mode of delivery at birth [1]. Of these factors, diet is considered to be a key modulator; hence, some macro- and micro-nutrients that mediate healthy metabolic homeostasis by modulating the growth of gut microbiota have gathered scientific attention [2,3,4]. Dietary fiber and prebiotic oligosaccharides have been extensively studied because substrates for gut fermentation are fundamentally non-digestible dietary components. Highly digestible food proteins have been considered to have little influence on the gut microbiota profile. However, a small amount of non-digestible proteins and peptides may reach the large intestine and be used by the microbiota as carbon and nitrogen sources [5,6]. Accumulating evidence suggests that dietary proteins can drive microbiota composition and function, and microbiota–protein interactions may significantly affect the host’s long-term health [5,6].

Cow milk is an important dietary ingredient that contains proteins, lipids, sugars, and minerals. Caseins comprise 80% of milk proteins and, based on the homology of the amino acid sequences, they are classified into α_s1_-, α_s2_-, β-, and κ-casein. There is a growing amount of interest in the function of milk from cows with genetic variants of β-casein [7,8]. Based on a point mutation in the amino acid at position 67, β-casein is described as A1 and A2 variants. The proline residue at this position in A2 β-casein is substituted with histidine in A1 β-casein. This difference in amino acid sequence makes A1 β-casein more susceptible to hydrolysis by proteolytic enzymes in the intestinal lumen. Many studies have demonstrated that the consumption of A1 β-casein can exacerbate gut environments and exert gut inflammation and diarrheic symptoms [7,8].

As the outcome of A1 versus A2 milk consumption has been debated [9,10], further research examining whether there are functional differences between A1 and A2 casein variants is warranted. Most studies to date have only compared the A1 and A2 casein variants in milk, and a few have compared their functions with other food proteins. In this study, we examined the effects of consuming A1 and A2 caseins on gut microbiota and fermentation in mice. Two types of casein were prepared from the milk of cows genotyped as A1A1 and A2A2, and their modulatory effects on gut microbiota were compared with those of mixed casein (commercial casein), soy protein isolate, and egg white. Mixed casein can be regarded as a reference protein because studies using rodent models often use mixed casein according to the recommendation of the American Institute of Nutrition [11]. Soy is a representative plant-based protein, and many studies examining the effect of dietary protein sources use soy rather than casein. From various animal proteins, this study selected eggs, a non-meat animal protein similar to milk casein. The objective was to determine whether A1 and A2 caseins can affect the gut microbiota and their metabolites and how these differences could be assessed compared to other food proteins.

## 2. Materials and Methods

### 2.1. Animals and Diets

A rodent model experiment was conducted using female C57BL/6 mice purchased from Charles River Laboratories Japan Inc. The mice were individually housed in plastic cages in a room maintained at 22±1 °C. The diet was formulated (per kg) with 200 g protein, 444.5 g corn starch, 150 g sucrose, 50 g lard, 50 g rapeseed oil, 40 g mineral mixture (AIN-93G-MX), 10 g vitamin mixture (AIN-93-VX), 3 g L-cystine, 2.5 g choline bitartrate, and 50 g cellulose. Soy protein isolate (Fuji Oil Co. Ltd., Osaka, Japan), mixed casein (Oriental Yeast Co. Ltd., Tokyo, Japan), and egg white (Nacalai Tesque Inc., Kyoto, Japan) were commercially available and used for diet formulation without further processing. The procedures and protocols for the animal experiments were approved by the Animal Care and Use Committee in accordance with the guidelines of the Advanced Science Research Center, Okayama University (OKU-2020856).

A1 and A2 caseins were prepared from Jersey cow milk. Exon 7 regions of the *CSN2* gene were amplified and sequenced using genomic DNA purified from the buffy coat [12]. Using the milk of cows genotyped as A1A1 and A2A2, caseins were fractionated via conventional isoelectric precipitation (pH 4.6, 25 °C). Most of the milk lipids were removed via centrifugation during fractionation, whereas the lipids remaining in the freeze-dried precipitates were removed by diethyl ether extraction. The lipid-free, dried particles were milled to obtain powdered A1 and A2 casein.

The experiment started at five weeks of age, and mixed casein, A1 casein, A2 casein, soy, and egg white were provided to groups of five mice. After feeding for four weeks, the mice were euthanized by administering anesthesia, followed by blood collection from the posterior vena cavae. The cecum contents were collected to analyze the short-chain fatty acid (SCFA) concentration and to extract the bacterial DNA, which was subsequently used for 16S rRNA gene amplicon sequencing [13].

### 2.2. Lysozyme Activity Assay

The rate of lysis of *Micrococcus lysodeikticus* was determined [14]. Egg white was dissolved at 0.5 mg/mL, and dried *M. lysodeikticus* cells were suspended at 0.15 mg/mL in 0.1 M potassium phosphate buffer (pH 6.25). An egg white solution and *M. lysodeikticus* suspension were mixed at 1:25, and the absorbance at 450 nm was monitored in kinetic mode for 10 min. One unit was defined as a linear decrease in absorbance of 0.001 per minute. The lysozyme activity of the egg white was 2500 units/mg.

### 2.3. Cecum SCFA Analysis

For SCFA determination, the cecum contents were homogenized with phosphate-buffered saline and deproteinized using 50 g/L metaphosphoric acid. The supernatant was used for the analysis of the SCFA concentration using a gas–liquid chromatograph (GC-14A; Shimadzu, Kyoto, Japan) fitted with a glass capillary column (15 m × 0.53 mm) coated with modified polyethylene glycol terephthalic acid (GL Sciences, Tokyo, Japan). The temperature of the column oven was set at 80 °C for the first 2 min, and then increased to 200 °C at a rate of 10 °C/min.

### 2.4. Bacterial DNA Extraction and 16S rRNA Gene Amplicon Sequencing

Bacterial DNA was extracted from 50 mg of cecum content using the repeated bead-beating plus column method [15]. A mini DNeasy stool kit (Qiagen, Germantown, MD, USA) was used to purify the DNA, which was then amplified via a two-step polymerase chain reaction (PCR) to generate amplicon libraries for next-generation sequencing. Primers targeting the V4 region of the 16S ribosomal RNA (rRNA) genes (forward:5′-ACACTCTTTCCCTACACGACGCTCTTCCGATCTGTGCCAGCMGCCGCGGTAA-3′; reverse:5′-GTGACTGGAGTTCAGACGTGTGCTCTTCCGATCTGGACTACHVGGGTWTCTAAT-3′) were used for the first round of PCR. PCR products were purified by electrophoretic separation on a 2.0% agarose gel using a FastGene^®^ Gel/PCR Extraction Kit (Nippon Genetics Co., Ltd., Tokyo, Japan). The second round of PCR was performed using adapter-attached primers. Second-round PCR products were purified in the same manner as in the first round. The purified amplicons were pair-end sequenced (2 × 250) on the Illumina MiSeq platform at FASMAC Co., Ltd. (Kanagawa, Japan). All sequencing data were received as FASTQ files and deposited in the NCBI Sequence Read Archive under the BioProject accession (PRJDB15437).

### 2.5. Bioinformatics

Bioinformatic analysis was performed using the QIIME2 program [16]. Raw paired-end FASTQ reads of bacteria and fungi were demultiplexed using the q2-demux plugin. Primer sequences were removed from the demultiplexed bacterial sequence data for quality control. DADA2 was used to filter, trim, denoise, and merge data. Chimeric sequences were removed using the consensus method. For phylogenetic diversity analysis, all observed amplicon sequence variants (ASV) were aligned using the MAFFT program plugin via q2-alignment to construct a phylogenetic tree with FastTree2 through q2-phylogeny. Taxonomic classification was assigned using the SILVA database (version 132), specific for the V3-4 region of the bacterial 16S rRNA gene. All taxonomic classifications were implemented using QIIME2 and assigned using a naïve Bayesian algorithm. α-diversity indices (Chao 1 and Shannon) were estimated using q2-diversity at the ASV level.

### 2.6. Statistical Analyses

Data on the metabolites (SCFA and ammonia-N level) and relative abundances of the cecum microbiota were subjected to the non-parametric Kruskal–Wallis sum-rank test. Dunn–Bonferroni pair-wise comparison was used for the post hoc test. The β-diversity of bacterial microbiota was quantitatively described using principal coordinate analysis (PCoA) biplots constructed according to the Bray–Curtis distance. The effect of dietary protein on β-diversity was tested using PERMANOVA with 999 permutations, and the taxa correlated with β-diversity at > 0.7 of a Pearson correlation coefficient were plotted as discriminant vectors. β-diversity analysis was performed using Primer 7 (ver. 7, Primer-E, Plymouth Marine Laboratory, Plymouth, UK).

## 3. Results

**Figure 1:**
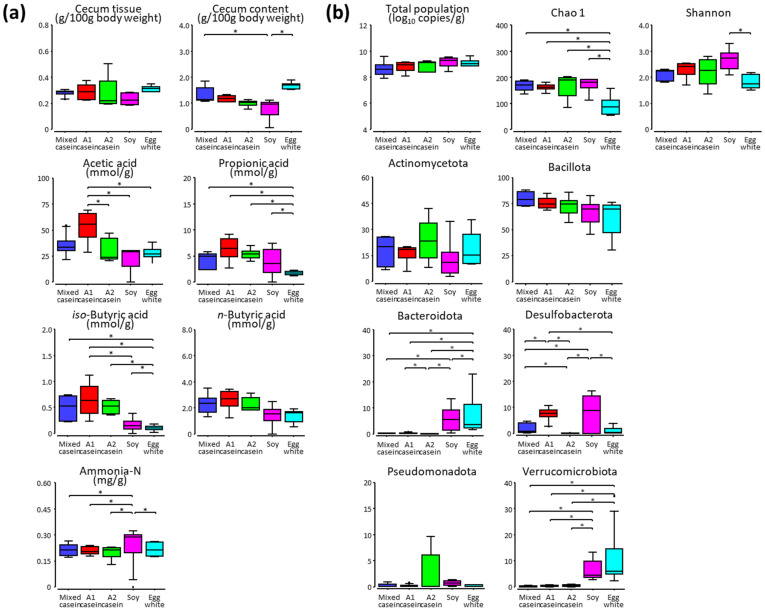
Differences in the metabolite levels and composition of the cecum microbiota in mice fed mixed casein, A1 casein, A2 casein, soy, and egg white for four weeks. The wet weights and metabolite levels (**a**) and total bacterial populations, alpha diversity indices, and relative abundances (%) of the major phyla (**b**) are indicated. The asterisk denotes a significant difference at *p* <0.05.

The cecum content (wet weight) was lower in mice fed soy than in those fed with the other proteins (Table S1; Figure 1a). Although no differences were observed in the cecum content between mice fed mixed A1 and A2 caseins, the acetic acid level was higher in A1 casein-fed mice than in A2 casein-fed mice. The levels of other SCFAs and ammonia-N were similar among the mice fed the three caseins. The propionic acid level was lower in mice fed egg white than in those fed other proteins, and the *iso*-butyric acid level was lower in mice fed soy and egg white than in those fed the three caseins. In contrast, ammonia-N levels were higher in mice fed soy than in those fed with other proteins.

The total population of cecum bacteria was approximately 9.0 log copies/g, regardless of the dietary proteins (Table S1; Figure 1b). However, the Chao 1 and Shannon indices were substantially lower in mice fed egg white than in those fed with other proteins. No differences were found in the total population and α-diversity of the cecum microbiota among the three casein diet groups.

Bacillota was the most abundant phylum, and Actinomycetota was the second most abundant phylum in the cecum microbiota, regardless of dietary proteins (Table S1; Figure 1b). The levels of Bacteroidota and Verrucomicrobiota were higher in mice fed soy and egg white than in those fed the three caseins, and the level of Desulfobacterota was higher in mice fed A1 casein and soy than in those fed mixed casein, A2 casein, and egg white. Although the abundance of Bacteroidetes and Desulfobacterota differed between A1 casein and A2 casein diets, the differences between the three caseins, soy, and egg white were greater.

**Figure 2:**
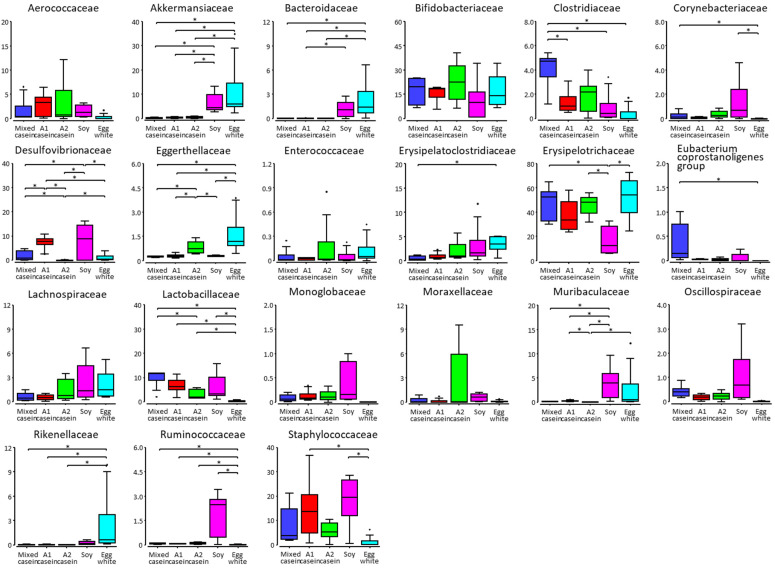
Relative abundances (%) of the major families of the cecum microbiota in mice fed mixed casein, A1 casein, A2 casein, soy, and egg white for four weeks. The families with a relative abundance of > 1% in at least one sample are indicated. The asterisk denotes a significant difference at *p* < 0.05.

Many taxa exhibited differences owing to dietary proteins at the family level (Table S2; Figure 2). The abundances of Eggerthellaceae, Muribaculaceae, and Desulfovibrionaceae differed between A1 casein-fed and A2 casein-fed mice. However, the abundance of Desulfovibrionaceae was high in mice fed soy in addition to in mice fed A1 casein, and the abundances of Eggerthellaceae and Muribaculaceae were much higher in mice fed egg white than in those fed A1 and A2 caseins. Mice fed mixed casein exhibited intermediate abundance levels between those of the mice fed A1 and A2 caseins for most of the taxa. The exceptions were Lactobacillaceae and Clostridiaceae; mice fed mixed casein demonstrated higher abundances than those fed A1 and A2 caseins.

**Figure 3:**
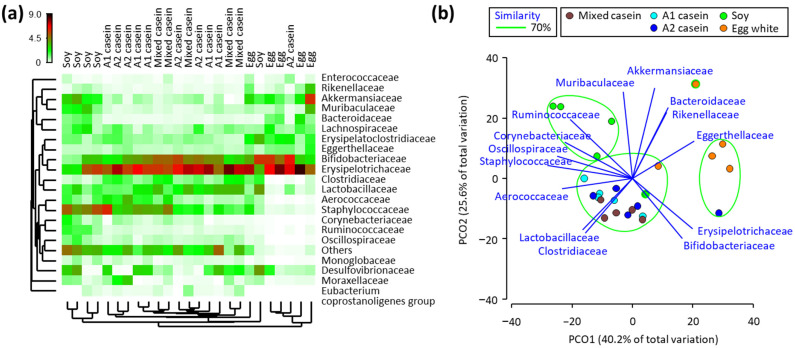
Configuration of the cecum microbiota in mice fed mixed casein, A1 casein, A2 casein, soy, and egg white for four weeks. Heatmap illustrating the hierarchical clustering (**a**) and principal coordinate plot characterizing the cecum microbiota (**b**) are indicated. The samples enclosed by a green line belong to the same group at 70% of the similarity level. The families with Pearson’s correlation > 0.7 are overlaid on the plot as vectors.

Distinctive microbiota were found in mice fed soy and egg white (Figure 3a). The abundances of Coryneabcteriaceae, Muribaculaceae, Desulfovibrionaceae, Staphylococcaceae, Lachnospiraceae, Monoglobaceae, Oscillospiraceae, and Ruminococcaceae were the highest in soy-fed mice. The abundances of Eggerthellaceae, Bacteroidaceae, Rikenellaceae, Erysipelatoclostridiaceae, Erysipelotrichaceae, and Akkermansiaceae were highest in egg white-fed mice.

The differences and similarities in the cecum microbiota between mice fed milk, soy, and egg proteins were demonstrated via PCoA (Figure 3b). The microbiota of mice fed mixed casein, A1 casein, and A2 casein formed one group, which was characterized by high abundances of Lactobacillaceae and Clostridiaceae and low abundances of Akkermansiaceae, Bacteroidaceae, and Rikenellaceae. The mice fed soy had high abundances of Corynebacterium, Staphylococcaceae, Oscillospiraceae, and Ruminococcaceae and low abundances of Bifidobacteriaceae and Erysipelotrichaceae. The mice fed egg white had high abundances of Akkermansiaceae, Bacteroidaceae, Rikenellaceae, and Eggerthellaceae and low abundances of Lactobacillaceae, Clostridiaceae, and Aerococcaceae.

## 4. Discussion

It has become common knowledge that dietary proteins can affect the gut microbiota and its metabolites [5,6,17,18,19,20]. We previously examined the microbiota of rats fed milk (mixed casein), soy, meat, eggs, and fish proteins [13,21,22]. The finding in this study, that egg protein feeding increased the cecum content and reduced the α-diversity of the microbiota, agreed with other studies, which could be partially accounted for by the antibacterial action of lysozyme in egg white [6,19]. However, the increased cecum content and reduced SCFA levels observed together may differ from the generally accepted findings, where an enlarged caecum content was observed with increased metabolite levels [13,21,22]. Thus, the effect of egg white feeding requires further evaluation by eliminating retained water based on the dry weight. The mice fed egg white seemed to adapt the cecum microbiota to the antibacterial substance by diluting the cecum content. Although Sivixay et al. [13] reported enhanced acetic acid levels due to soy feeding compared with casein feeding, this study found the highest acetic acid levels as a result of A1 casein feeding.

Despite many critical and objective opinions regarding the function of A1 and A2 caseins, the finding that the consumption of A1 milk may extend the digesta transit time, which could increase the risk of loose stool, appears to be widely accepted [10,23]. In this study, A1 casein-fed mice exhibited the highest total SCFA levels; hence, prolonged digesta transit may have stimulated the metabolic activity of the cecum microbiota. Dietary protein sources in this study did not affect the stool structure and frequency.

Although *iso*-butyric acid levels in the gut are considered to be related to the branched-chain amino acid content in the diet [24], the contents were reported to be similar for milk, soy, and egg proteins [25]. Thus, lowered *iso*-butyric acid levels in soy-fed mice may indicate a reduction in amino acid catabolism in the cecum microbiota. Meanwhile, higher ammonia-N levels were observed in soy-fed mice, possibly because of the lower digestibility of plant proteins than that of animal proteins, suggesting a sufficient level of microbiota activity. The curtailed cecum content with soy feeding compared with that of mixed casein feeding differed from the usual observation, which showed no differences between the two protein sources [19,20]. However, the total bacterial populations in the cecum content were similar regardless of the dietary proteins.

The abundance of Desulfovibrionaceae, an acetic acid producer, was the most distinctive difference between A1 and A2 casein feedings. Although the greater abundance in A1 casein-fed mice than that in A2 casein-fed mice in this study appears to be reasonable, Guantario et al. [26] reported the opposite result, i.e., that A2 casein feeding enhanced the abundance of Desulfovibrionaceae. Moreover, although Liu et al. [27] found that Ruminococcaceae and *Lactobacillus animalis* characterized the gut microbiota of A2 casein-fed mice, Ruminococcaceae characterized the microbiota of soy-fed mice in this study. Furthermore, although Lactobacillaceae and Clostridiaceae characterized the microbiota of mice fed the three caseins in this study, Zhu et al. [18] and Xia et al. [19] reported reductions in these taxa with casein feeding. Thus, although dietary proteins can be considered to be modulators of the gut microbiota, the modulation patterns may not be stably demonstrated. In this study, only the SCFA and ammonia-N levels were determined to evaluate the effects of dietary proteins. Members of Desulfovibrionaceae are sulfate-reducing bacteria that produce H_2_S, which is detrimental at an excessive concentration to the resolution of gut inflammation [28]. Further studies should examine other metabolites, such as H_2_S, indole, skatole, and amines, to ascertain the difference between A1 and A2 casein feedings.

The abundance of Muribaculaceae also differed between mice fed A1 and A2 caseins. Muribaculaceae, a family formerly referred to as S24-7, is one of the major mucin monosaccharide foragers, which include Bacteroidaceae, Bifidobacteriaceae, Akkermansiaceae, and Ruminococcaceae [29]. Except for Bifidobacteriaceae, these taxa were more abundant in mice fed soy and egg white than in those fed three caseins. Thus, although casein feeding may lower the abundance of mucin degraders, the difference between A1 and A2 casein feedings was substantially smaller than that found between soy, egg white, and the three casein feedings. Although the abundance of Muribaculaceae in feces has been reported to correlate with the propionic acid level [30], the relationship was not observed in the cecum content in this study.

Increased abundances of Eggerthellaceae, Rikenellaceae, and Erysipelotrichaceae with egg white feeding appear to be reproducible [14]. The antibacterial action of lysozyme may explain the changes caused by egg white feeding [20]. Many Gram-positive taxa (Corynebacteriaceae, Lactobacillaceae, Staphylococcaceae, Clostridiaceae, Monoglobaceae, Oscillospiraceae, Ruminococcaceae, and the Eubacterium coprostanoligenes group) exhibited the lowest abundances, and many Gram-negative taxa (Bacteroidaceae, Muribaculaceae, Rkenellaceae, and Akkermansiaceae) were the most abundant in mice fed egg white. However, several Gram-positive taxa (Eggerthellaceae, Erysipelatoclostridiaceae, and Erysipelotrichaceae) were detected at the highest abundances after egg white feeding. Eggerthellaceae is known to be able to convert the isoflavone daidzein into equol [31]; hence, the taxon could be enhanced in soy-fed mice. The abundance of Eggerthellaceae was higher in mice fed egg white and A2 casein than in those fed soy and A1 casein. Thus, although the effect of lysozyme may be substantial, the actions of dietary proteins and antibacterial components may be complicated. The abundance of Erysipelotrichaceae is shown to be positively linked to a lipidemic imbalance and hypercholesterolemia [32].

The increased abundance of Akkermansiaceae found in mice fed egg white can be considered a health-associated change because the level of *Akkermansia muciniphila* has been shown to be positively correlated with the parameters involved in fatty acid oxidation and inversely associated with inflammatory markers [33,34]. Sivixay et al. [13] found an increase in the taxon in rats fed meat rather than egg white; hence, changes in the level of this Gram-negative taxon due to egg white feeding require further investigation.

This study used young female C57BL/6 mice because juvenile animals may be susceptible to dietary interventions. Many factors, such as age, sex, and strain, remain unexplored and need to be clarified in further studies. The mice were fed until nine weeks of age (around puberty); hence, sex differences could not be marked because differences in the gut microbiota between males and females may become apparent at middle age after puberty [35]. Strain differences between C57BL/6, BALB/c, and NOD mice were demonstrated by the abundance of Lactobacillus and Akkermansia [36]. Thus, even when mice are used for assessment, several strains with putatively different baseline gut microbiota may be better used. Furthermore, the limitations of rodent model studies should be considered when one is interpreting the findings [36]. However, the differences between soy, egg, and milk proteins were greater than those between A1 and A2 milk proteins, which could be extrapolated to humans to some extent. Our findings should be considered when assessing and discussing the functionality of A1 and A2 milk.

## 5. Conclusions

The microbiota and fermentation in the cecum were affected by A1 casein and A2 casein feeding, and the abundance of Desulfovibrionaceae and the acetic acid level were higher in A1 casein-fed mice than in A2 casein-fed mice. However, most bacterial taxa of the cecum microbiota were similar between mixed casein, A1 casein, and A2 casein feedings, and the β-diversity demonstrated by PCoA plots indicates the same group for mice fed the three caseins. Changes in the microbiota due to soy and egg proteins were distinctive for many bacterial taxa. The α-diversity of the gut microbiota was significantly lower in mice fed egg white than in those fed caseins and soy. Although differences can be seen between A1 casein and A2 casein regarding modulatory effects on the gut microbiota and metabolites, soy, egg, and other proteins may also affect gut environments; hence, the functional evaluation of A1 and A2 caseins should explore various protein sources.

## Data Availability

Raw data are stored in private computers and are available upon request.

## Supplementary Materials

The following supporting information can be downloaded at: https://www.mdpi.com/article/10.3390/microorganisms11051194/s1, Table S1: Wet weight, metabolites level, and microbiota composition of the cecum in mice fed mixed casein, A1 casein, A2 casein, soy protein isolate, and egg white for four weeks; Table S2: Microbiota of the cecum in mice fed mixed casein, A1 casein, A2 casein, soy protein isolate, and egg white for four weeks; Table S3: Results of PERMANOVA using a Bray-Curtis distance matrix to examine differences in the cecum microbiota of mice fed mixed casein, A1 casein, A2 casein, soy, and egg white.
