# Supplementary material for: Modulatory Effects of A1 Milk, A2 Milk, Soy, and Egg Proteins on Gut Microbiota and Fermentation
Source: Microorganisms. 2023 May 3;11(5):1194. doi: 10.3390/microorganisms11051194 (PMC10223512; doi:10.3390/microorganisms11051194)
Supplement: Supplementary file 1 [file microorganisms-11-01194-s001.zip › microorganisms-2302558-supplementary final.pdf]

Table S1. Wet weight, metabolites level, and microbiota composition of the cecum in mice fed mixed casein, A1 casein, A2 casein, soy protein isolate, and egg white for four weeks.

	**Mixed casein**	**A1 casein**	**A2 casein**	**Soy**	**Egg white**	**SE**
Cecum weight (g/100g body weight)						
Tissue	0.27	0.29	0.29	0.24	0.36	0.04
Content	1.34 [a]	1.18 [ab]	1.00 [ab]	0.73 [b]	1.53 [a]	0.13
Short-chain fatty acids (μmol/g)						
Acetic acid	35.6 [ab]	53.2 [a]	31.1 [b]	27.5 [b]	27.9 [b]	5.21
Propionic acid	4.18 [a]	6.37 [a]	5.37 [a]	4.89 [a]	1.72 [b]	0.79
iso-Butyric acid	0.49 [ab]	0.65 [a]	0.51 [a]	0.21 [bc]	0.10 [c]	0.10
n-Buryric acid	2.28	2.59	2.30	1.76	1.35	0.32
Total SCFAs	42.6 [ab]	62.8 [a]	39.3 [ab]	34.3 [b]	31.1 [b]	6.05
Ammonia-N (μg/g)	215 [b]	210 [b]	198 [b]	292 [a]	217 [b]	16.2
Total population (log_{10} copies/g)	8.63	8.80	8.86	9.15	9.10	0.21
Alpha diversity						
Chao 1	168 [a]	162 [a]	164 [a]	170 [a]	90.6 [b]	15.9
Shannon index	2.10 [ab]	2.28 [ab]	2.18 [ab]	2.67 [a]	1.83 [b]	0.18
Phylum (%)						
Actinomycetota	17.5	15.9	23.9	13.0	19.1	4.87
Bacteroidota	0.22 [cd]	0.34 [bc]	0.12 [d]	5.75 [ab]	7.59 [a]	2.12
Desulfobacterota	1.98 [b]	7.47 [a]	0.06 [c]	7.79 [a]	1.20 [b]	1.75
Bacillota	79.9	75.6	72.5	66.1	60.5	5.63
Pseudomonadota	0.28	0.22	2.94	0.71	0.20	0.90
Verrucomicrobiota	0.17 [b]	0.36 [b]	0.53 [b]	6.61 [a]	11.3 [a]	2.87

Mean values for five mice. Values in the same row with unlike superscript letters are significantly different based on the non-parametric Kruskal–Wallis sum-rank test ($p<0.05$).

Table S2. Microbiota of the cecum in mice fed mixed casein, A1 casein, A2 casein, soy protein isolate, and egg white for four weeks

	**Mixed casein**	**A1 casein**	**A2 casein**	**Soy**	**Egg white**	**SE**
Family (%)						
Bifidobacteriaceae	17.0	15.5	22.7	11.2	17.5	5.03
Corynebacteriaceae	0.24^{a}	0.06^{ab}	0.36^{ab}	1.43^{a}	0.01^{b}	0.40
Eggerthellaceae	0.24^{b}	0.30^{b}	0.82^{a}	0.28^{b}	1.61^{a}	0.29
Bacteroidaceae	0.04^{bc}	0.03^{c}	0.04^{bc}	1.20^{ab}	2.25^{a}	0.57
Muribaculaceae	0.13^{bc}	0.26^{b}	0.05^{c}	3.94^{a}	2.80^{ab}	1.32
Rikenellaceae	0.04^{b}	0.04^{b}	0.02^{b}	0.23^{ab}	2.52^{a}	0.85
Desulfovibrionaceae	1.98^{b}	7.47^{a}	0.06^{c}	7.79^{a}	1.20^{b}	1.75
Erysipelatoclostridiaceae	0.57^{b}	0.95^{ab}	2.11^{ab}	3.29^{ab}	3.40^{a}	1.15
Erysipelotrichaceae	46.9^{a}	37.4^{ab}	45.6^{a}	16.9^{b}	52.0^{a}	6.48
Aerococcaceae	1.77	2.83	3.44	1.58	0.34	1.33
Enterococcaceae	0.06	0.02	0.18	0.05	0.12	0.09
Lactobacillaceae	9.73^{a}	6.65^{ab}	3.15^{b}	6.24^{ab}	0.23^{c}	1.72
Staphylococcaceae	8.39^{ab}	14.5^{a}	5.68^{ab}	18.0^{a}	1.27^{b}	4.11
Clostridiaceae	4.06^{a}	1.34^{b}	1.85^{ab}	0.91^{b}	0.38^{b}	0.60
Lachnospiraceae	0.65	0.55	1.51	2.56	2.19	0.75
Monoglobaceae	0.09	0.13	0.13	0.41	0.00	0.10
Oscillospiraceae	0.42	0.19	0.23	1.09	0.01	0.27
Ruminococcaceae	0.08^{a}	0.06^{a}	0.10^{a}	1.83^{a}	0.01^{b}	0.30
Eubacterium coprostanoligenes group	0.38^{a}	0.03^{ab}	0.02^{ab}	0.06^{ab}	0.00^{b}	0.09
Moraxellaceae	0.25	0.15	2.87	0.58	0.09	0.88
Akkermansiaceae	0.17^{b}	0.36^{b}	0.53^{b}	6.61^{a}	11.3^{a}	2.87

Mean values for five mice. Values in the same row with unlike superscript letters are significantly different based on the non-parametric Kruskal–Wallis sum-rank test (p<0.05).

Table S3. Results of PERMANOVA using a Bray-Curtis distance matrix to examine differences in the cecum microbiota of mice fed mixed casein, A1 casein, A2 casein, soy, and egg white.

Transform: Square root
Resemblance: S17 Bray-Curtis similarity
Sums of squares type: Partial
Permutation method: Unrestricted permutation of raw data
Number of permutations: 999

PERMANOVA table of results

Source	df	SS	MS	Pseudo-F	P(perm)	
Protein	4	7885.6	1971.4	4.8737		0.001
Residual	20	8090	404.5			
Total	24	15976				

Pair-wise tests

Groups	t	P(perm)	perms	
Casein, A1		1.2315	0.244	126
Casein, A2		1.3211	0.151	126
Casein, Soy		2.0395	0.029	126
Casein, Egg		3.0751	0.007	126
A1, A2		1.5956	0.033	126
A1, Soy		1.639	0.048	126
A1, Egg		3.1508	0.008	126
A2, Soy		2.0252	0.024	126
A2, Egg		2.3345	0.018	126
Soy, Egg		2.4769	0.012	126
